# Genetic Syndromes and Genes Involved in the Development of the Female Reproductive Tract: A Possible Role for Gene Therapy

**DOI:** 10.4172/2157-7412.1000127

**Published:** 2013

**Authors:** MT Connell, CM Owen, JH Segars

**Affiliations:** 1 Department of Obstetrics and Gynecology, Truman Medical Center, Kansas City, Missouri; 2 Department of Obstetrics and Gynecology, University of Pennsylvania School of Medicine, Philadelphia, Pennsylvania; 3 Program in Reproductive and Adult Endocrinology, Eunice Kennedy Shriver National Institute of Child Health and Human Development, National Institutes of Health, Bethesda, Maryland, USA

**Keywords:** Müllerian anomalies, Vaginal anomalies, Genetic syndromes, Gene therapy, HOX genes, Mayer-Rokitansky-Kuster-Hauser syndrome (MRKH)

## Abstract

Müllerian and vaginal anomalies are congenital malformations of the female reproductive tract resulting from alterations in the normal developmental pathway of the uterus, cervix, fallopian tubes, and vagina. The most common of the Müllerian anomalies affect the uterus and may adversely impact reproductive outcomes highlighting the importance of gaining understanding of the genetic mechanisms that govern normal and abnormal development of the female reproductive tract. Modern molecular genetics with study of knock out animal models as well as several genetic syndromes featuring abnormalities of the female reproductive tract have identified candidate genes significant to this developmental pathway. Further emphasizing the importance of understanding female reproductive tract development, recent evidence has demonstrated expression of embryologically significant genes in the endometrium of adult mice and humans. This recent work suggests that these genes not only play a role in the proper structural development of the female reproductive tract but also may persist in adults to regulate proper function of the endometrium of the uterus. As endometrial function is critical for successful implantation and pregnancy maintenance, these recent data suggest a target for gene therapy. Future research will be needed to determine if gene therapy may improve reproductive outcomes for patients with demonstrated deficient endometrial expression related to abnormal gene expression.

## Introduction

The first investigations into abnormal female reproductive tract development began centuries ago, however with the progress of modern molecular genetics only now are the underlying mechanisms of this complex process becoming elucidated. Several genes have been identified in the abnormal and normal development of the uterus, cervix, fallopian tubes, and vagina. Many anomalies are felt to be multifactorial; however there are case reports of familial inheritance suggesting that specific genetic mutations may cause these defects [1]. Furthermore, there are defined genetic syndromes that feature anomalies of the female reproductive tract.

Additionally, animal models have helped identify candidate genes involved in the development of these anomalies and cases of discordant monozygotic twins have also provided insight into the possible involvement of epigenetic mechanisms [2]. The most common of the Müllerian anomalies are uterine anomalies, which may be associated with either adverse or normal reproductive outcomes depending on the variant. In most cases of uterine anomalies, adverse outcomes are related to difficulty in maintaining pregnancy rather (e.g. recurrent pregnancy loss, late first or second trimester loss) than conceiving (e.g. primary infertility) [3, 4]. Tis review will cover the normal embryologic development of the female reproductive tract, important genes involved in normal development, as well as genetic mutations associated with abnormal development and genetic syndromes featuring Müllerian and vaginal anomalies. Finally, a review of recent research demonstrating that continued adult expression of genes is critical to normal embryologic development suggests a role for gene therapy as a possible treatment modality for clinical sequelae of Müllerian anomalies in the future.

## Normal Development of the Female Reproductive Outflow Tract

While distinctly separate from the urinary system, the genital system is linked with the urinary system in the embryological stages of development [5]. The urogenital system develops into the kidneys, gonads, and the urinary and reproductive tracts. The Wolffian (mesonephric) and Müllerian (paramesonephric) ducts are the primordia of the male and female reproductive tracts, respectively. Gonadal development is a separate developmental process that is determined by the sex chromosomes. Absence of anti-Müllerian hormone (AMH, normally produced by the male testes) will trigger stabilization of the Müllerian system and regression of the Wolffian system leading to development of the female reproductive tract. Normal development of the fallopian tubes, uterus, and upper vagina requires an intricate progression of Müllerian duct elongation, fusion, canalization, and septal resorption [6]. The Müllerian ducts originate from coelomic epithelium of the lateral walls of the urogenital ridge by week 6 of embryonic development. The Müllerian ducts elongate caudally and fuse in the midline crossing the Wolffian ducts to form a Y-shaped structure, which is the primordium of the uterovaginal canal [7]. The cranial aspects of the Müllerian ducts remain open; as this forms the abdominal ostium of the oviduct. The urogenital sinus fuses with the caudal end of the fused Müllerian ducts by week 10 of development. Subsequent canalization of the Müllerian ducts results in two channels with a mid-line dividing septum. Typically by week 20, resorption of the septum in a caudal to cephalad direction has been completed.

The vaginal plate originates from proliferation of the sinovaginal bulbs which result from fusion of the Müllerian ducts and urogenital sinus. At approximately 20 weeks of development, the lumen of the lower vagina is then created as cells at the center of the vaginal plate degenerate in a caudal to cephalad direction. The hymenal membrane, whose central epithelial cells typically degenerate prior to birth, is the division of the vaginal lumen and the urogenital sinus. As evidenced above, there are many intricate steps involved in normal development of the female reproductive tract and any failures of the described processes may result in congenital anomalies.

## Genetic Mechanisms Regulating Development of the Female Reproductive Tract

The female reproductive tract is essential for the continuation of the human species. A range of developmental defects including agenesis, atresia, and septation of the reproductive tract, many of which have been associated with genetic syndromes, have been documented [8]. Despite the important effect of anomalies on reproduction, the molecular and cellular mechanisms that govern its normal and abnormal formation are incompletely understood [9]. Most knowledge of genes regulating development arises from human genetic syndromes that affect the female reproductive tract or mouse knockout studies; both of which have helped identify key genes in the development of this organ system [10-12]. In the sections to follow, candidate genes critical to the development of the female reproductive tract are reviewed as well as genetic syndromes that feature Müllerian anomalies.

### Genes responsible for Müllerian duct formation and diferentiation

**Figure 1:**
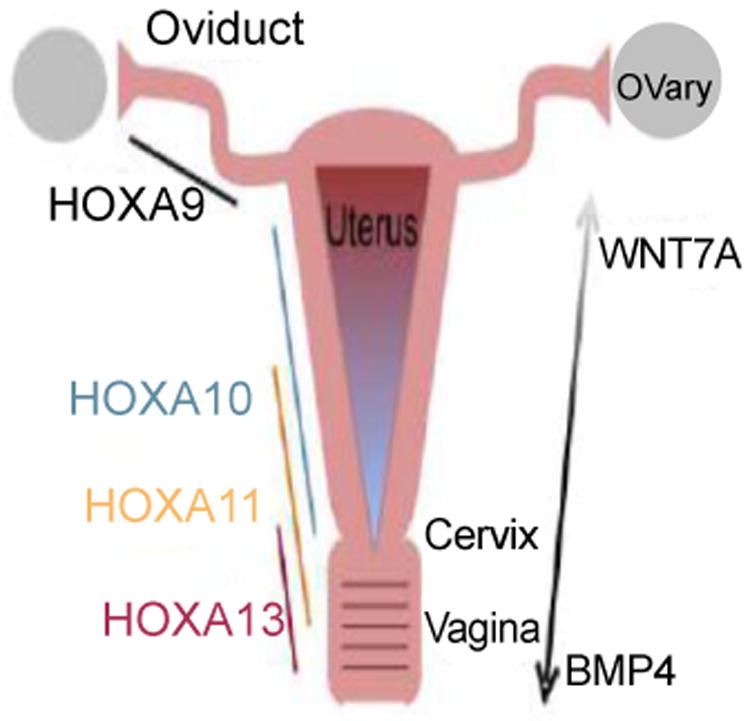
Schematic Diagram of Gene Expression in Development of Female Reproductive. **Tract:** Tissue specific regulation by the Hoxa family homeobox transcription factors are important in the development in the oviducts (Hoxa9), uterus (Hoxa10 and Hoxa11), cervix (Hoxa11 and Hoxa13) and vagina (Hoxa11 and Hoxa13) as shown. Wnt family genes are believed to be involved in the anterior-posterior as well as radial patterning. Specifcally shown here is Wnt7a, which is required for maintenance of Hoxa10 and Hoxa11 expression in the uterus. Also illustrated is the gradient of BMP4 expression, strongest in the vagina and weakest in the uterus. The opposite gradient has been noted for Wnt7a

Knock out mouse models have provided insight into the signaling molecules and transcription factors essential for Müllerian duct formation [10-12]. Development of the Müllerian ducts is considered a triphasic process consisting of initiation, invagination, and elongation [7]. Phase one involves coelomic epithelial cells being specified for a Müllerian duct fate. Mechanisms controlling this first phase have not been fully elucidated, however specification of cells can be recognized by the presence of Lim homeobox 1 (Lim1). Lim1 is key to the development of the Müllerian duct epithelium as absence of Lim1 in mice leads to a phenotype lacking oviducts, uterus, and an upper vagina [13]. Following initiation, invagination occurs through the expression of wingless-type MMTV integration site family, member 4 (WNT4) [13]. This gene is known to antagonize the testis-determining factor and play a critical role in both the control of female development and the prevention of testes formation. This second phase ends when the invaginating Müllerian ducts contact the Wolffian ducts. The first two phases have been shown to be Wolffian duct independent [14]. On the contrary, the third phase of elongation requires the maintenance of the Wolffian ducts [15]. Conditional inactivation of Lim1 has been associated with Wolffian duct regression and subsequently results in incomplete development of the Müllerian ducts [14]. The elongation phase also involves proliferation of cells at the mesoepithelial tip of the Müllerian ducts which requires the presence of the Wolffian ducts through Wnt9b signaling which is associated with regulation of cell fate and patterning during embryogenesis [7, 16]. Paired box 2 (Pax2) is another gene that has been shown necessary for the second phase of Mullerian duct development. Knock out mouse models for Pax2 lack both a genital tract and kidneys in male and female animals [17]. It has been demonstrated in these knock out mice that coelomic epithelium invaginates, however the Wolffian ducts degenerate and hence the Müllerian ducts do not elongate leading to failure of the third phase [17]. Empty spiracles homeobox 2 (Emx2) is expressed in epithelial components of the urogenital system and absence of Emx2 leads to a complete absence of the urogenital system, which is obviously essential for normal development of the female reproductive outflow tract [18]. Emx2 mutant mice show abnormal expression of Lim1, Pax2, and Wnt4 in the intermediate mesoderm [18]. These related mechanisms suggest an underlying genetic pathway for the formation of the Müllerian ducts. Roles for the previously mentioned genes have been mostly elucidated; however Müllerian duct formation is not completely understood and other key genes in the pathway continue to be discovered. Retinoic acid appears to be involved in the anterior posterior patterning and in female reproductive tract development but few details are known. In the mouse model, compound mutations of retinoic acid receptors either demonstrate absence of the entire female reproductive tract or only the caudal portions [19, 20]. The POU domain-containing transcription factor 2 (Tcf2) gene has been shown in mouse models to be expressed during the earliest steps of female reproductive tract formation [21]. Mutations of TCF2 in humans have been associated with bicornuate and didelphic uteri [22]. Similar abnormalities have been described in discs, large homolog 1 (Dlgh1) null mice, who experience aplasia of the cervix and vagina from failed lateral fusion of the Müllerian ducts [23]. Transcriptional cofactors dachshund homolog 1 and 2 (Dach1 and Dach2) seem to fit within this developmental cascade as the double knock out mouse model shows complete failure to develop Müllerian duct derivatives [24]. Following Müllerian duct formation, differentiation occurs along an antero-posterior (A-P) and radial axis. This includes the formation of the oviducts, uterus, cervix, and vagina. This occurs through interactions between the Müllerian duct epithelium and the surrounding mesenchyme [25]. This A-P patterning establishes histologically distinct segmental boundaries. The anterior boundary occurs between the oviduct and uterine body, and the posterior boundary is between the uterus and cervix [25]. This patterning is primarily regulated by Hoxa family homeobox transcription factors. Hoxa9, Hoxa10, Hoxa11, and Hoxa13 are expressed uniformly along the A-P axis (Figure 1). Hoxa9 is expressed in the oviduct whereas Hoxa10 and Hoxa11 are expressed in the uterus. Hoxa11 and Hoxa13 can be found in the cervix and anterior vagina [26]. Hoxa10 and Hoxa11, as expected, are required for patterning and differentiation of the uterus and their expression patterns overlap during embryogenesis [27]. For example, Hoxa10 mutants have demonstrated homeotic transformation of the anterior part of the uterus into oviduct like structures leading to reduced fertility [28]. Hoxa11 has been shown to be necessary for proper organization of uterine stroma where loss of this gene leads to thinner, shorter uteri and no endometrial glands suggesting a more anterior phenotype [11, 29]. On the contrary, an alternative study demonstrated absence of uterosacral ligaments (USL) in Hoxa11 null mice suggesting a more posterior phenotype [30]. Hoxa13 null mice show agenesis of the distal portion of the Müllerian ducts indicating a role for Hoxa13 not only in differentiation but also in the formation of Müllerian ducts [31]. Temporal and spatial variation in expression of the Hoxa family genes may explain the diversity of uterine shapes seen that perhaps result from different degrees of Müllerian duct fusion [31]. Several genes aside from the Hoxa family genes have also been shown to regulate Müllerian duct differentiation. Wnt family genes appear to control the A-P and radial patterning. Wnt7a null mutant mice exhibit several abnormalities including shortened and uncoiled oviducts, hypoplastic uterine horns, and a vaginal septum [32]. In addition, Wnt7a null mutant mice have been shown to have endometrial gland agenesis, disorganized myometrium, a reduction in the stromal compartment of the uterus and posterior appearing uteri [32]. In these mice, the posterior aspect of the oviduct resembles the uterus, and the uterus has similar characteristics to the vagina [32]. Wnt7a appears to be required for the maintenance of Hoxa10 and Hoxa11 as knockout of Wnt7a has shown decreased expression of Hoxa10 and Hoxa11 [32]. Wnt5a also appears to be important in this pathway as null mice die at birth secondary to improper A-P axis development [33]. Elegant grafting models have allowed for more precise study of the role of Wnt5a. Wnt5a mutant mice were shown to have short, coiled uterine horns; but lack defined cervical and vaginal structures [33]. This phenotype is similar to that of Hoxa13 mutants. Wnt5a mutants were also shown to have absent uterine glandular formation [33]. Both knock out models of Wnt5a and Wnt7a suggested an important role in glandular genesis. Both Wnt5a and Wnt7a are required for correct glandular genesis as these genes are expressed in the uterine stroma and uterine epithelium respectively [32, 33]. In areas where uterine epithelial invaginations occur, Wnt7a was down regulated. Wnt5a appears to be critical in this down regulation leading to endometrial glandular formation [33]. This highlights the role of epithelial-mesenchymal interaction required for uterine development [34]. As noted above, knockout of Wnt5a was shown to be associated with glandular agenesis, however the luminal epithelium was noted to be intact [33]. Catenin (cadherin-associated protein), beta 1 (Ctnnb1) produces the protein β-catenin and is a downstream effector of the Wnt family genes. Knock out of this protein leads to absence of uterine glandular tissue and an epithelium that resembles that of the vagina [35]. Finally, forkhead box A2 (Foxa2) has been identified as an important regulatory gene in gland formation as ablation of Foxa2 leads to glandular agenesis [36]. The exact factors that interact upstream or downstream of Foxa2 are not currently known, but no change in Wnt5a or Wnt7a was observed in this ablation model [36]. However, Foxa2 expression was noted to be absent in the Wnt7a conditional knockout, suggesting that Foxa2 is downstream of Wnt7a [37]. The pathways that orchestrate Müllerian duct formation and differentiation are obviously complex. Several of the underlying genetic mechanisms have been described, but more research is needed to gain a fundamental understanding of the genetic basis of the female reproductive tract.

### Genes responsible for development of the Vagina

Controversy remains surrounding the developmental origins of the vagina. It has been a commonly held belief (based on murine studies) that the vagina is of dual origin. The Müllerian ducts form the cranial portion, the so-called “Müllerian vagina”, while the urogenital sinus is the origin of the caudal portion, the so-called “sinus vagina” as reviewed by Cai [38] and Kurita [39]. However, based on murine studies Cai [38] concluded that the recent evidence supports a Müllerian duct origin for the entire vagina. More recent cell lineage tracing studies in humans have demonstrated that the Wolffian ducts, Müllerian ducts, and the urogenital sinus all play integral roles during the formation of the human vagina in a caudal to cranial manner [40]. The authors showed that cells of the urogenital sinus (staining positively for p63, a marker of squamous epithelium) migrated via the open Wolffian ducts to reach the caudal tips of the fused Müllerian ducts where proliferation of the cells formed the vaginal primordium [39, 40]. Based on these findings, the authors hypothesized that the fused Müllerian ducts only contributed as the guiding structure for the developing vagina, rather than a cellular origin as previously suggested. The intricate genetic mechanisms controlling this process remain incompletely understood.

## Müllerian Vagina

In murine models, Hoxa13 and bone morphogenetic protein 4 (BMP4) appear to be strongly expressed in the Müllerian vagina but not in the uterus [26, 38, 41]. In these murine models, Hoxa13 has been shown to up regulate BMP4 [42]. BMP4 appears to control the ventral mesodermal fate of the mesodermal primordium, while concurrently Wnt7a controls the dorsal signals [43, 44]. Animal models have shown that BMP4 disrupts Wnt7a signaling [45]. A gradient of BMP4, strongest in the vagina and weakest in the uterus, has been demonstrated with the opposite gradient noted for Wnt7a (expressed in Mullerian epithelium). It has also been noted that Wnt7a subsequently disappears in the vagina when BMP4 appears [46]. It is believed that BMP4 is responsible for desensitizing the mesoderm to anti-Müllerian hormone and inducing a stratified squamous cell in the vagina [38]. Importantly, Wnt7a drives the expression of anti-Müllerian hormone receptor type II, which is responsible for Müllerian regression [47]. Thus BMP4 disrupts the Wnt7a signal making the primordium insensitive to anti-Müllerian hormone. BMP4, as previously noted, is also responsible for the differentiation of the vaginal epithelium to stratified squamous cells through activation of p63, also known as cytoskeleton-associated protein 4 [48, 49]. p63 has numerous roles in the formation of the lower female reproductive tract including the differentiation from Müllerian epithelium to vaginal squamous epithelium, cloacal septation, and external genital modeling [50, 51]. p63 null mice show cloacal abnormalities, and human genetic syndromes with associated genital anomalies have had p63 mutations identified [50, 52].

## Sinus Vagina

The so-called sinus vagina is also formed through the influence of BMP4 expression in the surrounding mesenchyme. This influence occurs before Hoxa13 is present. At this point in development, Sonic Hedgehog (SHH) has been shown to be the activator of BMP4 guiding the Müllerian ducts caudally and playing a key role in the partitioning the cloaca [38]. Knockout animal models of SHH have shown improper partitioning of the cloaca [53].

## Müllerian and Vaginal Anomalies in Genetic Syndromes affecting Humans

With that background, our focus will now turn to genetic syndromes affecting the female genital tract in humans. Much of the knowledge gathered from the murine models discussed thus far has been used to discern the genetic causes of these anomalies. In this section we will review select disorders, their familial aggregates, genetic mechanisms, and the available molecular data.

### Vaginal atresia

Vaginal atresia is a rare condition. Its occurrence is 1:4000 to 1:10, 000 females [54]. Vaginal atresia is most often characterized by absence of the hymen, and occasionally by absence of the vagina extending to the cervix. Physical exam of affected patients reveals normal Müllerian structures, including the cervix, uterus, and oviducts; but the vagina is replaced by fibrous tissue [1]. Familial aggregates of isolated vaginal atresia have not been reported in the literature. Therefore, it has been challenging to determine the developmental origins of this rare condition. However, there are several genetic syndromes that have been identified with vaginal atresia as a commonly associated malformation (Table 1). McKusick-Kaufman (MKKS) and Bardet-Biedl (BBS) are two autosomal recessive syndromes with significant overlap which have been associated with vaginal agenesis [1, 55-58]. In females, MKKS is typically characterized by congenital heart malformations, postaxial polydactyl, and hydrometrocolpos. Hydrometrocolpos, a fluid filled dilated vagina and uterus, can be caused by obstruction from vaginal atresia, transverse vaginal septa, or an imperforate hymen. BBS is associated with all of the MKKS anomalies plus visual impairment, developmental delays, and obesity. Hence a suspected diagnosis of MKKS made in the neonatal period may evolve to be BBS if developmental delays, visual impairment, and obesity become apparent [59]. The MKKS gene, responsible for the MKKS phenotype, has been mapped to chromosome 20p12 and has been associated with chaperone protein folding, processing, and assembly [59]. The MKKS gene has been identified in one sub-type of BBS, while several BBS gene family members as well as 10 other genes have been associated with the BBS phenotype [60]. The proteins encoded by these genes, which are structurally diverse, share roles in cilia formation and function [60]. No genes have been identified that are associated with isolated vaginal atresia.

### Transverse vaginal septum

Transverse vaginal septa occur in about 1 per 75, 000 females [61]. It is believed that transverse vaginal septa result from failure of the urogenital sinus and Müllerian ducts to fuse and canalize. Typically the superior aspect of the septum contains columnar epithelium classic of Müllerian epithelium. Conversely, the inferior aspect contains squamous epithelium typical of the urogenital sinus. This distinction in epithelium fuels the controversy over the developmental origin of the vagina, and supports the notion of a dual developmental origin. In the case of a transverse vaginal septum, the pelvic organs and lower vagina are typically normal [1]. However, we have encountered a case of bladder exstrophy with an upward anteriorly displaced bicornuate uterus and an associated incomplete transverse vaginal septum. With our understanding of the developmental pathway of the female reproductive tract, this association of anomalies suggests a genetic syndrome, which we refer to as DeCherney syndrome. To our knowledge, this is the first description of this association. Genetic syndromes previously discussed (Table 1) may feature transverse vaginal septa instead of vaginal atresia. The question, as with vaginal atresia, remains as whether or not similar genes are involved in isolated and syndromic cases of transverse vaginal septa [1]. Aside from the MKKS gene and the genes of the BBS family, there are no candidate genes for isolated transverse vaginal septa. Discovering the underlying genetic mechanisms leading to the formation of transverse vaginal septa would undoubtedly add to our understanding of the developmental origins of the vagina and perhaps resolve the controversy.

### Longitudinal vaginal septum

A longitudinal vaginal septum may be in the coronal or most often, the sagittal plane. The diagnosis of longitudinal vaginal septum should be distinguished clinically from incomplete Müllerian fusion. Incomplete Müllerian fusion defects may extend caudally to produce a vaginal septum [1]. A few genetic syndromes and case reports have been identified with longitudinal vaginal septum. Camptobrachydactyly presents with brachydactyly, polydactyly, urinary incontinence, and longitudinal vaginal septa [62]. Johanson-Blizzard syndrome has been associated with multiple anomalies including longitudinal vaginal septa. In the first description of this syndrome a septate vagina was noted, however the report did not discuss the possibility of incomplete Müllerian fusion [1]. The gene for Johanson-Blizzard syndrome has been identified as ubiquitin protein ligase E3 component n-recognin 1 (UBR1), but the genetic mechanisms leading to the known phenotype are not fully understood as the gene is generally known to be involved in a proteolytic pathway of the ubiquitin system [63]. A third syndrome, hand-foot-genital syndrome is characterized by hand defects, urinary tract anomalies, and Müllerian ducts anomalies [64]. The Müllerian defects range from vaginal septa to uterine didelphys [64]. Hand- foot-genital syndrome is an autosomal dominant condition with known mutations in HOXA13 [65]. As with vaginal atresia and transverse vaginal septa, currently only genes associated with genetic syndromes have been identified in the case of longitudinal vaginal septa. The underlying basic genetic mechanisms leading to formation of longitudinal vaginal septa remain to be determined.

### Imperforate hymen

Imperforate hymen occurs in about 1 in 1000 females [61]. This condition is most often described as the absence of the central portion of the hymen. In cases of imperforate hymen leading to obstruction, vaginal and/or uterine distention with fluid may ensue (also known as hydrocolpos or hydrometrocolpos). As previously discussed, transverse vaginal septa or vaginal atresia may cause similar symptoms, however the presence of vulvar distension is uniquely suggestive of an imperforate hymen [1]. While most cases of imperforate hymen are sporadic, inherited cases have been described. McIlroy and Ward [66] as well as Usta et al. [67] have described cases of affected siblings suggesting an autosomal recessive inheritance. Stelling et al. [68] described a family of concordant monozygotic twins with one of the twins having an affected daughter suggesting an autosomal dominant inheritance. Some rare syndromes have been described which are characterized by imperforate hymen. Ulnar-mammary syndrome is associated with upper limb structures, apocrine/mammary hypoplasias, dental abnormalities, and genital anomalies including imperforate hymen [69]. Alterations in the TBX3 gene, a downstream target of retinoic acid, have been implicated as the cause of ulnar-mammary syndrome [70]. As discussed earlier, perturbations in retinoic acid leads to posterior defects in the female reproductive tract [19], thus giving a possible genetic explanation as to the cause of hereditary imperforate hymen in the ulnar-mammary syndrome. Unfortunately, no other candidate genes associated with imperforate hymen have been identified other than TBX3.

## External Genital Anomalies

Little is understood regarding the genetic mechanisms leading to external genital anomalies including congenital fusion of the labia, complete absence of the labia, and various cloacal malformations. Congenital fusion of the labia is typically associated with virilization from congenital adrenal hyperplasia [71]. However, cases of familial labial fusions not associated with adrenal hyperplasia have been reported [71]. Absence of the labia, has been reported in popliteal pterygium syndrome [1]. Cloacal malformations, as previously mentioned, have been linked to mutations in p63. Loss of p63 does not allow for the urorectal septum to form leading to a persistent cloaca [50]. Knockout mice for p63 exhibit hypoplastic genitals and a single cloacal opening [50]. In humans the genetic syndrome of ectrodactyly, ectodermal dysplasia, and facial clefts (ECC) results from p63 mutations [52]. ECC consists of a multitude of developmental anomalies that include genital anomalies [52]. Patients with cloacal anomalies should be tested for p63 mutations.

### Atresia of the Cervix

Several case reports of congenital absence of the cervix exist [72]. The cervix may be absent in lieu of a normal uterus and vagina; however, some cases have reported atresia of the vagina and cervix suggesting close developmental origins of these structures [1, 72]. To our knowledge there are no reported cases of familial cervical agenesis and no candidate genes have been identified to date.

### Incomplete Müllerian fusion

Incomplete Müllerian fusion is difficult to estimate as many patients are asymptomatic; however, the incidence is quoted as between 0.1-3% [61]. Traditionally, incomplete Müllerian fusion results in two hemiuteri each associated with one fallopian tube, but various forms of incomplete Müllerian fusion exist. Incomplete Müllerian fusion represents a range of conditions from a complete hemiuteri to an atreitic rudimentary horn [73]. There have been several case reports of familial aggregates of incomplete Müllerian fusion [1]. Several syndromes have been indentified with incomplete Müllerian fusion as a common component (Table 2). Contrary to the cases of vaginal atresia and vaginal septa, there have been several isolated or nonsyndromic cases of incomplete Müllerian fusion identified for study leading investigators to look for candidate genes in these cases. HOXA10 and HOXA 11, previously discussed as being expressed in the uterus, have been investigated. Liatsikos et al. [74] examined 30 women with Müllerian defects. Only one patient was found to have a mutation in HOXA10, however her mother had a similar mutation but was notably phenotypically normal [74]. Cheng et al. [75] conducted a mutation analysis in 109 Chinese women with Müllerian anomalies and found one mutation (Y57C) was found in HOXA10 that affected the gene's ability to induce and repress other genes. This mutation was isolated in the affected patient's father as well. Wang et al. [76] found a rare variant in the PAX2 gene in a patient with uterus didelphys reinforcing the role of PAX2 in Müllerian duct development. Other candidate genes which have been investigated include PBX1, WNT7A, and LHX1. However, none have been linked with incomplete Müllerian fusion [77-79]. Some candidate genetic mutations have been reported in association with incomplete Müllerian fusion, but further research is needed as much is still unknown.

### Müllerian aplasia

Müllerian Aplasia is the absence of the uterus, cervix, and upper vagina. The lower third of the vagina is typically present secondary to normal formation of the sinus vagina and the external genitalia are normal. On occasions uterine remnants may be present. Most often the diagnosis of Müllerian aplasia is labeled as Mayer-Rokitansky-Kuster-Hauser (MRKH) syndrome [1]. MRKH may feature isolated Müllerian aplasia (type 1), or be associated with renal, skeletal, auditory, and cardiac defects (type II) [2]. The presence of other associated anomalies suggests that an initial insult to the intermediate mesoderm leads to alteration of the cervicothoracic somites and the pronephric ducts [80]. Most cases are sporadic; however several reports of familial clustering are suggestive of a genetic cause [81]. These cases of familial clustering appear to occur by autosomal dominant inheritance with incomplete penetrance and variable expressivity [82]. Investigations based on these cases have subsequently been directed to certain candidate genes [2]. AMH was one of the first genes to be investigated because of its role in regression of the Müllerian ducts. However, no abnormal expression of AMH or activating mutation of its associated receptor has been found in studies to date [83]. WT1 and PAX2, discussed earlier, have been identified as significant in early embryonic development; however, there have been no mutations in these genes linked to MRKH [84-86]. Notably, despite the association between MRKH with both galactosemia and cystic fibrosis, studies found no link between mutations in GALT and CFTR genes and the MRKH phenotype [87, 88]. Investigations into Hoxa10, Hoxa11, and analysis of PBX1, a cofactor of HOX genes, have failed to determine a cause of Müllerian aplasia [74, 78]. Subsets of MRKH, especially cases associated with genetic syndromes, have been attributed to specific genetic etiologies. WNT4 mutations are associated with Müllerian aplasia, hyperandrogenism, and renal malformations [89]. In this phenotype, failure to suppress androgens in the ovary occurs leading to Müllerian aplasia [90, 91].

Mutations in HNF1β (also known as TCF2, previously discussed) have been associated with maturity-onset diabetes of the young renal dysfunction and Müllerian aplasia [92]. WNT7A mutations have recently been recognized as causing Al-Awadi/Raas-Rothschild/Schinzel phocomelia syndrome; which is characterized as having several limb deformities and uterine hypoplasias/aplasia [93]. Other syndromes listed in table 3 are associated with Müllerian aplasia. While some candidate genes have mutations known to cause MRKH, very few such mutations have been found. The recent advent of high-resolution array based studies has led to newly recognized variants at certain chromosome loci that harbor genes of interest in Müllerian aplasia [94]. Nik-Zainal et al. [94] identified three microdeletions at 16p11.2, 17q12, 22q11.2 that were significantly enriched when the syndromic Müllerian aplasia case population was compared to the control population. Nested in the 16p11.2 location, TBX6 was identified as an important gene in paraxial mesoderm development [94]. Nested in the 17q12 location are HNF1β and LHX1 (also known as LIM1, previously discussed) [94]. Ledig et al. [95] found recurrent deletions affecting TBX6, HNF1β, and LHX1 in their cohort of MRKH patients. Four other genes; RTDR1, RAB36, GNAZ, and BCR; were identified in the 22q11.2 location; however, no mutations in these genes have been described in humans [94]. Microdeletions at these loci had been previously described in association with other congenital malformations involving spine, genitourinary tract, and the cardiovascular system [94]. Duplication in the SHOX gene has recently been identified in two daughters with MRKH type I and their phenotypically normal father [96]. Microdeletions in 1q21.1 have also been found most often associated with thrombocytopenia-absent radius syndrome (TAR) [97]. The incidence of uterine and genital abnormalities is estimated to be 6% of cases of TAR syndrome. A few cases of TAR with associated MRKH in females have been described in the literature, but this is a rare occurrence [58, 98, 99]. The above chromosome loci and genes discussed may partially explain the familial and syndromic cases of Müllerian aplasia; however, most cases remain sporadic [82]. In one retrospective review of women with Müllerian aplasia undergoing IVF via a surrogate, no cases of Müllerian anomalies were reported in the reproductive offspring suggesting no dominant inheritance pattern [100]. Recent case reports of discordant monozygotic twins have suggested the involvement of epigenetic factors in MRKH [101]. Recently, Rall et al. [2] analyzed 8 MRKH patients and 8 controls using whole-genome expression and methylation. The authors found that the estrogen receptor 1 (ESR1), Wilms Tumor 1 (WT1), and GATA binding protein 4 (GATA4) had increased expression in MRKH patients [2]. This was a significant finding given that WT1 and GATA4 play significant roles in male sex differentiation via regulation of AMH and that estrogen has been reported to regulate AMH [102, 103]. The authors hypothesized that the increased expression of the genes might lead to increased AMH promoter activity during development and subsequent Müllerian duct regression leading to a MRKH phenotype [2]. Gene expression clearly plays a significant role in development, and recently microRNAs have been shown to be important for post-transcriptional regulation of gene expression in the development and function of the female reproductive tract [104]. Conditional mouse knockouts of DICER1, an important RNAse for proper formation of miRNAs, demonstrated shortened uterine horns and oviductal diverticuli [105]. In these mice, 28 miRNAs were found to be downregulated [105]. Of the identified 28, 23 miRNAs were predicted to target previously mentioned important candidate genes such as Wnt5a, Hoxa9, and Hoxa10 [105]. This suggests an important role for miRNAs in the development and function of the female reproductive tract.

## Endocrine Disruptors of Müllerian Duct Development

The developing female reproductive tract is highly sensitive to synthetic hormones and exposure to these substances in utero can lead to the development of Müllerian anomalies [9]. Some investigators have used these endocrine disruptors to help determine the genetic basis of development of the female reproductive tract. Diethylstilbestrol (DES), a synthetic form of estrogen, has been shown to downregulate Wnt7a, Hoxa10, and Hoxa11 through activation of ESR1 [106, 107]. Post DES exposure, HOXA9 expression is shifted from the oviducts to the uterus while HOXA10 and HOXA11 expression in the uterus is decreased causing the characteristic ‘T-shaped’ uterus that resembles the human phenotype [2, 9, 108-110]. Clearly a greater understanding of endocrine disruptors in the environment and their role in the development of Müllerian defects may help elucidate the underlying genetic mechanisms.

## Reproductive Outcomes and a possible role for Gene Therapy in patients with Müllerian

### Anomalies

Despite the relative rarity of Müllerian anomalies, they can significantly impact reproductive outcomes usually affecting the maintenance of pregnancy rather than conception [3, 4]. As previously stated, the most commonly encountered are uterine anomalies that occur in approximately 3-4% of women (despite fertility), 5-10% of women with recurrent pregnancy loss, and up to 25% of women with preterm delivery or late first or second trimester loss [4, 6, 111-113]. Understanding the underlying genetic mechanisms of abnormal development of the female reproductive tract may help us understand these adverse outcomes. It has been recently suggested that Hox family genes may not simply be important to the understanding of our embryologic development, but may also serve a regionally specific regulatory role in the adult female reproductive tract [114]. For example in adult mice, Hoxa10 expression in the endometrium has been shown to be critical for successful implantation and subsequent fertility [115-117]. This begs the question, could alterations in HOX gene expression in the endometrium of humans explain implantation and maintenance of pregnancy problems seen in humans affected by Müllerian anomalies? Rackow et al. [118, 119] have demonstrated that in the case of both submucosal uterine fibroids and endometrial polyps, both acquired structural abnormalities of the uterine cavity known to adversely affect reproductive outcome, that HOX gene expression is globally decreased in the uterus of affected individuals compared to controls. These significant findings indicate an ongoing role for HOX gene expression in the normal physiological changes of the adult. It is unclear whether the ‘adult’ functions are a continuance of the original embryonic pathways, or if there are new patterns of gene expression regulated by HOX in the adult [114].

Many patients affected by Müllerian anomalies may have normal reproductive outcomes; however, intervention is recommended in the event of adverse obstetric outcomes. Surgical intervention may be necessary for many of these patients (i.e. septate uterus), but perhaps there is role for gene therapy to increase HOX expression in the other patients with uterine anomalies and poor reproductive outcomes that are at this time difficult to explain. Future research should be aimed at determining whether HOX gene expression is altered in patients with Müllerian anomalies and if this expression demonstrates an association with reproductive outcomes.

## Conclusions

Recent focus on the genetic basis of female reproductive tract malformations has provided insight into the underlying molecular mechanisms that govern this process crucial to the survival of our species. Several genes thought to play a significant role in this developmental pathway have been identified by analysis of knock out mouse models and through identification of genetic syndromes that feature anomalies of the female reproductive tract. Müllerian anomalies can significantly impact reproductive outcomes; therefore, future studies should focus on continuing to uncover the underlying genetic and molecular mechanisms of the development of the female reproductive tract as many of these embryologically defined genes may have a role in adult reproductive functions. In the not to distant future, perhaps gene therapy could be used to target these genes with maintained expression in the adult to change reproductive outcomes for patients.

## Figures and Tables

**Table 1:** Syndromes associated with vaginal atresia.

Syndromes	Somatic Findings	Reproductive Anomaly	Etiology
Antley-Bixler	Craniosynostosis, choanal atresia, radiohumerus synostosis, gracile ribs, camptodactyly, renal defects	Vaginal Atresia	Autosomal Dominant FGFR2 mutation Autosomal Recessive POR mutation
Apert	Craniosynostosis and midface hypoplasias with syndactyly of hands and feet, cardiac and renal defects	Vaginal Atresia	Autosomal Dominant FGFR2 gene mutation
Bardet-Biedl	Mental retardation, pigmentary retinopathy, polydactyly, obesity, hypogonadotropic hypogonadism	Vaginal Atresia	Autosomal Recessive BBS1-14 mutations
del(1)(q12)	Growth and mental retardation, facial anomalies, neural tube defects, absence of corpus callosum	Vaginal Stenosis	Chromosomal
Ellis Van Creveld	Congenital heart defects, short limbs, postaxial polydactyly	Vaginal Atresia	Autosomal Recessive EVC/EVC2 mutations
Fraser	Cryptophalamos, nose and ear anomalies, laryngeal stenosis, renal agenesis, mental retardation	Vaginal Atresia	Autosomal Recessive FRAS1, FREM2, GRIP1 mutations
McKusick-Kaufman	Hydrometrocolpos, postaxial polydactyly, cardiac defects, esophageal atresia, anal atresia	Vaginal Atresia	Autosomal Recessive MKKS mutations
Pallister Hall	Hypothalamic hamartoblastoma, panhypopituitarism, craniofacial defects, postaxial polydactyly, renal and cardiac defects	Vaginal Atresia	Autosomal Dominant GLI3 mutation
Robinow	Mesomelic dwarfism, hypertelorism, cleft lip and palate, anteverted nares, hemivertebra, short digits	Vaginal Atresia	Autosomal Dominant WNT5A mutation Autosomal Recessive ROR2 mutation

Listed here are genetic syndromes which may be associated with vaginal atresia. Other phenotypic characteristics of the genetic syndromes, modes of inheritance, and known genetic mutations are presented.

**Table 2:** Syndromes associated with incomplete müllerian fusion (IMF).

Syndromes	Somatic Findings	Uterine anomaly	Etiology
Acro-renal mandibular	Limb deficiencies, diaphragmatic hernia, ectrodactyly of hand and foot, absence of radius and metacarpal V, kidney dysplasia	Uterus didelphys	Autosomal recessive
Apert	Apert Craniosynostosis and midface hypoplasias with syndactyly of hands and feet, cardiac and renal defects	Bicornuate uterus	Autosomal Dominant FGFR2 gene mutation
Bardet-Biedl	Mental retardation, Pigmentary retinopathy, polydactyly, obesity, hypogonadotropic hypogonadism	“Uterus duplex, vaginal septa”	Autosomal recessive BBS1-14 mutations
Beckwith-Wiedemann	Omphalocele, macroglossia, overgrowth, clitoral enlargement	IMF	Imprinting abnormality, hypo-, hypermethylation of 11p15.5
Caudal duplication	Duplication of sacrum, lumbar vertebrae, anus, large bowel, external genitalia	Duplication of uterus and cervix	Unknown
Caudal regression	Agenesis of sacral and lumbar regions	Duplication of uterus and cervix	Unknown
Cloacal exstrophy	Common urogenital sinus and rectum, renal anomalies, vertebral defects	IMF	Unknown
de Lange	Growth retardation, microcephaly, mental retardation, synophrys, limb anomalies	IMF	Mutations in NIPBL, SMC1A, SMC3
Donohue	Elfin facies, enlarged ears, low-set ears, prominent breasts, abnormal carbohydrate metabolism, insulin receptor defect	IMF	Autosomal Recessive Insulin Receptor gene mutations
Female pseudohermaphroditism with renal and gastrointestinal anomalies	Genital ambiguity, urologic and gastrointestinal anomalies, vertebral and radial anomalies, renal absence	Uterine Didelphys	Unknown
Fraser	Cryptophthalmos, nose and ear anomalies, laryngeal stenosis, renal agenesis, mental retardation	Bicornuate uterus	Autosomal recessive FRAS1, FREM2, GRIP1 mutations
Fryns	Coarse facies, cleft palate, pulmonary hypoplasias, diaphragmatic defects	Bicornuate uterus	Autosomal recessive No gene identified yet
Halal	Digital hypoplasias, upper limb shortening, ectrodactyly	Uterine didelphys with	Autosomal Dominant Unknown gene
Hydrolethalus	Hydrocephaly, neural tube defects, micrognathia, deep set eyes, cleft palate, malformed respiratory tract, cardiac anomalies, club feet, polydactyly	“Uterus duplex”	Autosomal recessive KIF7 and HYLS1 mutations
Jarcho-Levin	Spondylocostal dysostosis: hemivertabrae, vertebral absences and fusion, respiratory defects, cardiac defects, short neck and chest, hernias	Uterine didelphys	Autosomal recessive MESP2 mutations
Meckel	Encephalocele, postaxial polydactyly, dysplastic polycystic kidneys, male pseudohermaphroditism	Bicornuate uterus	Autosomal recessive MSK1, TMEM216, TMEM67, CEP290, RPGRIP1L, CC2D2A mutations
Popliteal Pterygium	Pterygium of popliteal, antecubital, and crural regions, cleft lip and palate, digital hypoplasia	IMF	Autosomal Dominant IRF6 mutations Autosomal Recessive RIPK4
Roberts	Tetraphocomelia, craniofacial abnormalities, corneal clouding, cardiac and renal anomalies	Bicornuate uterus	Autosomal recessive ESCO2 mutations
Rüdiger	Mental retardation, coarse facies, bifid uvula, ureteral stenosis, thickened palms and soles, inguinal hernias, poor cartilaginous formation	Bicornuate uterus	Autosomal recessive Unknown gene
Thalidomide embryopathy	Tetraphocomelia, especially radius, tibia, femur, midline facial hemangioma, nerve palsies, and cardiac defects	Septate uterus and vagina	Teratogen
Urogenital adysplasia	Unilateral or bilateral renal agenesis, flattened facies, pulmonary hypoplasias, limb deformations	Unicornuate or Bicornuate uterus	Unknown: multifactorial, epigenetic inheritance

Listed here are genetic syndromes that may be associated with incomplete Müllerian fusion (IMF). Other phenotypic characteristics of the genetic syndromes, modes of inheritance, and known genetic mutations are presented.

**Table 3:** Syndromes associated with müllerian aplasia.

Syndromes	Somatic Findings	Uterine Anomaly	Etiology
Deletion 4p (Wolf-Hirschhorn syndrome)	Microcephaly, mental retardation, growth retardation, cardiac anomalies	Absent uterus	Chromosomal [del (4)(p16.3]
Oculoauriculovertebral spectrum (Goldenhar syndrome)	Hypoplastic malar, maxillary and mandibular regions, microtia, hemivertebrae or hypoplastic vertebrae	“Rokitansky sequence”	Unknown: multifactorial, epigenetic inheritance, environmental disruption
Female pseudohermaphroditism, renal and gastrointestinal anomalies	Genital ambiguity, urologic and gastrointestinal anomalies, vertebral and radial anomalies, renal absence	Absence of uterus	Unknown
Al-Awadi/Raas-Rothschild	Absence or reduction of limbs, facial abnormalities, pelvic and genital abnormalities	Müllerian aplasia	Autosomal recessive WNT7A Mutation
Müllerian aplasia, Klippel-Feil anomaly	Short neck, low hairline, restricted mobility of upper spine, middle ear anomalies	Müllerian aplasia	Unknown
MURCS association	Müllerian aplasia, renal aplasia, cervicothoracic somite dysplasia	Müllerian aplasia	Unknown
Roberts	Tetraphocomelia, craniofacial abnormalities, corneal clouding, cardiac and renal anomalies	Agenesis of uterus and agenesis or atresia of vagina	Autosomal recessive ESCO2 Mutation
Thalidomide embryopathy	Tetraphocomelia, midline facial hemangioma, nerve palsies, cardiac defects	Müllerian aplasia	Teratogen
Mosaic trisomy 7	Cystic kidneys, oligohydramnios	Absence of uterus	Chromosomal
Urogenital dysplasia (hereditary renal dysplasia)	Potter facies, Pulmonary hypoplasias, limb deformations, renal dysplasia	Absence of uterus	Unknown: multifactorial, epigenetic inheritance

Listed here are genetic syndromes that may be associated with Müllerian aplasia. Other phenotypic characteristics of the genetic syndromes, modes of inheritance, and known genetic mutations are presented.
